# Intermediate Megavoltage Photon Beams for Improved Lung Cancer Treatments

**DOI:** 10.1371/journal.pone.0145117

**Published:** 2015-12-16

**Authors:** Ying Zhang, Yuanming Feng, Munir Ahmad, Xin Ming, Li Zhou, Jun Deng

**Affiliations:** 1 Department of Biomedical Engineering, Tianjin University, Tianjin, China; 2 Department of Therapeutic Radiology, Yale University School of Medicine, New Haven, Connecticut, United States of America; 3 Department of Radiation Oncology, William W. Backus Hospital, Norwich, Connecticut, United States of America; 4 Center for Radiation Physics and Technology, West China Hospital Cancer Center, Sichuan University, Chengdu, China; North Shore Long Island Jewish Health System, UNITED STATES

## Abstract

The goal of this study is to evaluate the effects of intermediate megavoltage (3-MV) photon beams on SBRT lung cancer treatments. To start with, a 3-MV virtual beam was commissioned on a commercial treatment planning system based on Monte Carlo simulations. Three optimized plans (6-MV, 3-MV and dual energy of 3- and 6-MV) were generated for 31 lung cancer patients with identical beam configuration and optimization constraints for each patient. Dosimetric metrics were evaluated and compared among the three plans. Overall, planned dose conformity was comparable among three plans for all 31 patients. For 21 thin patients with average short effective path length (< 10 cm), the 3-MV plans showed better target coverage and homogeneity with dose spillage index R_50%_ = 4.68±0.83 and homogeneity index = 1.26±0.06, as compared to 4.95±1.01 and 1.31±0.08 in the 6-MV plans (p < 0.001). Correspondingly, the average/maximum reductions of lung volumes receiving 20 Gy (V_20Gy_), 5 Gy (V_5Gy_), and mean lung dose (MLD) were 7%/20%, 9%/30% and 5%/10%, respectively in the 3-MV plans (p < 0.05). The doses to 5% volumes of the cord, esophagus, trachea and heart were reduced by 9.0%, 10.6%, 11.4% and 7.4%, respectively (p < 0.05). For 10 thick patients, dual energy plans can bring dosimetric benefits with comparable target coverage, integral dose and reduced dose to the critical structures, as compared to the 6-MV plans. In conclusion, our study indicated that 3-MV photon beams have potential dosimetric benefits in treating lung tumors in terms of improved tumor coverage and reduced doses to the adjacent critical structures, in comparison to 6-MV photon beams. Intermediate megavoltage photon beams (< 6-MV) may be considered and added into current treatment approaches to reduce the adjacent normal tissue doses while maintaining sufficient tumor dose coverage in lung cancer radiotherapy.

## Introduction

Lung cancer has become the most common and deadly cancer in the world with estimated 1.59 million deaths each year, accounting for nearly one in five of the total cancer mortality worldwide [1]. In the United States, lung cancer accounts for more deaths than any other cancers in both men and women [2]. In recent years, stereotactic body radiation therapy (SBRT) has shown promising outcome in the radiotherapeutic management of early-stage non-small cell lung cancer (NSCLC) for inoperable lung cancer patients [3]. Despite improved local control and survival, SBRT approach for lung cancer still faces challenges in reducing radiation toxicity to the normal tissues.

Although 6-MV and higher megavoltage photon beams have been dominantly used in the clinic for decades, recently there has been growing interest in intermediate energy photon beams (< 6-MV). This is because intermediate energy photons have narrower penumbra due to reduced range of secondary electrons. Faster dose fall-off and lower exit dose can also benefit the adjacent critical structures. If multiple gantry angles are employed in plan optimization, good target coverage can also be achieved with intermediate energy photon beams without over-dosing the superficial tissues [4].

Several studies have been carried out recently involving intermediate energy photon beams. In 2007, Keller *et al*. showed that 1.2-MV x-rays combined with small fields can reduce the radiological penumbra in intracranial stereotactic radiosurgery (SRS), which could be substantially beneficial for improving target dose homogeneity and better sparing of critical structures [5]. Fox *et al*. have compared Cobalt-60 gamma-ray with 6/18-MV photons and demonstrated that nearly identical intensity-modulated radiotherapy (IMRT) plans can be achieved between Co-60 and 6-MV photons [6]. Later on, Stevens *et al*. configured a 4-MV flattening filter free (FFF) beam to improve the dose distribution at tissue-air interface for lung tumor treatment [7]. More recently, Dong *et al*. investigated a 2-MV FFF beam for extracranial robotic IMRT. Their results demonstrated that the dual energy plan (2- and 6-MV) had the best dosimetry in terms of equivalent target coverage and improved organs-at-risk (OAR) sparing, followed by 2-MV only and 6-MV only plans [4].

Mixed energies for cancer treatments have been investigated in the past [8–10]. In a study by St-Hilaire *et al*., beam energy was added as an optimization parameter in an automatic aperture-based inverse planning system [10]. Their work demonstrated that energy optimization using 6 and 23 MV beams could produce plans of better quality with less peripheral dose and fewer MUs for prostate and lung tumors. Park *et al*. investigated the effect of mixing 6-MV and 15-MV photon beams on prostate cancer IMRT treatments and concluded that mixed-energy plans have similar target coverage, improved OARs dose and integral dose for deep seated tumors [8].

3-MV photon beams have significantly lower energy than 6-MV photon beams with distinctly different beam characteristics. To our best knowledge, so far there has been no systematic study on the potential dosimetric effects of 3-MV photon beams on radiotherapy treatments of lung cancers. Hence, the aim of this study is to investigate the dosimetric effects of intermediate energy photon beams, particularly 3-MV photons on the lung SBRT treatments with IMRT. The dosimetric effects of mixed energy plan using intermediate energy photons (3-MV) and clinically widely used 6-MV photons was also explored.

## Materials and Methods

### Virtual Linac beam modeling and validation

In this study, a virtual 3-MV photon beam was modeled with Monte Carlo method based on a Varian linear accelerator (Varian Medical Systems, Palo Alto, CA). Specifically, an EGS4/BEAM Monte Carlo code has been used to simulate the particles emanating from a Varian Linac treatment head with nominal energy of 3-MV [11,12]. The geometry and the materials used in the EGS4/BEAM Monte Carlo simulation reflected a realistic construction of the Linac operating at 6 MV photon mode, only the energy of incident electron beam was set to be 3 MeV. Particularly, various component modules were constructed with the EGS4/BEAM Monte Carlo code to model the treatment head of the Linac including the target, primary collimator, exit window, flattening filter, monitor chamber, secondary collimator, jaws and protection window. A full phase space file was first scored above the photon jaws located at 28 cm downstream from the target. The phase space data contains multi-dimensional information for each particle across the chosen plane, including the position, direction, charge, energy, weighting factor, and a tag to record the particle history [13]. The full phase space can be sampled for further particle transport in the rest of the geometry. However, the large amount of information to be stored and the slow sampling speed during retrieval of all this information is the major limitation of the phase space approach [14]. As an alternative, multiple source models can be derived with EGS4/BEAMDP based on the phase space data [15]. The obtained multiple source models consisted of detailed numerical description of the energy spectrum, spatial distribution, fluence distribution, source location, shape and size of each source for a particular treatment head [15,16]. The multiple source models have been shown equivalent to the phase space data in representing the photon beams from the Linac treatment head and replicating the dose distributions in water, yet eliminating the inconvenience of large data transfer and latent variance related to the phase space [13,15,16].

The obtained multiple source models were then used as beam input in EGS4/MCSIM for Monte Carlo dose simulations so that all the required beam data such as depth doses and transverse dose profiles for various square and rectangular field sizes ranging from 3 cm × 3 cm to 40 cm × 40 cm were generated in a water phantom. Output factors normalized to 10 cm × 10 cm field size at 95 cm SSD and 5 cm depth in water were also calculated. For all the simulations, the EGS4 transport parameters were set as electron cut-off energy (ECUT) = AE = 700 keV and photon cut-off energy (PCUT) = AP = 10 keV. AE is the low-energy threshold for γ-ray production while AP is the low-energy threshold for soft bremsstrahlung production. The voxel size ranged from 0.25 cm × 0.25 cm × 0.25 cm in dose profile simulations to larger steps along depth direction in depth dose simulations. The calculation time of each Monte Carlo simulation was between 1 to 52 hours on a single CPU workstation in order to achieve a statistical uncertainty (1σ) of less than 2%. The benchmark results of EGS4/MCSIM have been reported previously [17,18].

**Fig 1:**
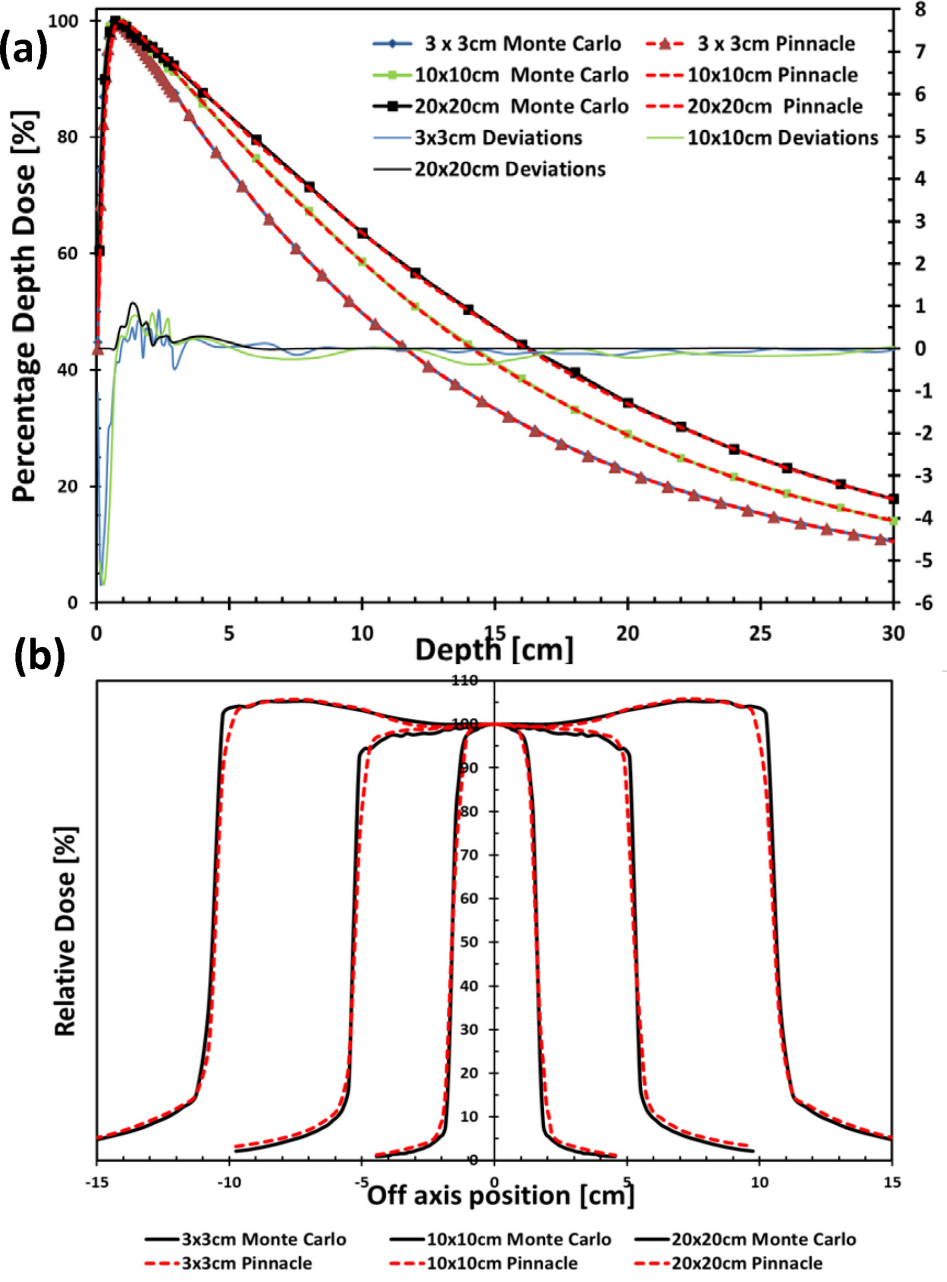
Comparison of (a) percentage depth dose (PDD) curves and (b) lateral dose profiles at 5 cm depth for field sizes of 3×3 cm^2^, 10×10 cm^2^ and 20×20 cm^2^ between Monte Carlo simulated (solid lines) and Pinnacle3 calculation results (dashed lines) for the 3-MV photon beam at 100 cm source-to-surface distance. The deviations in depth doses were shown in the lower part to the right scale.

**Fig 2:**
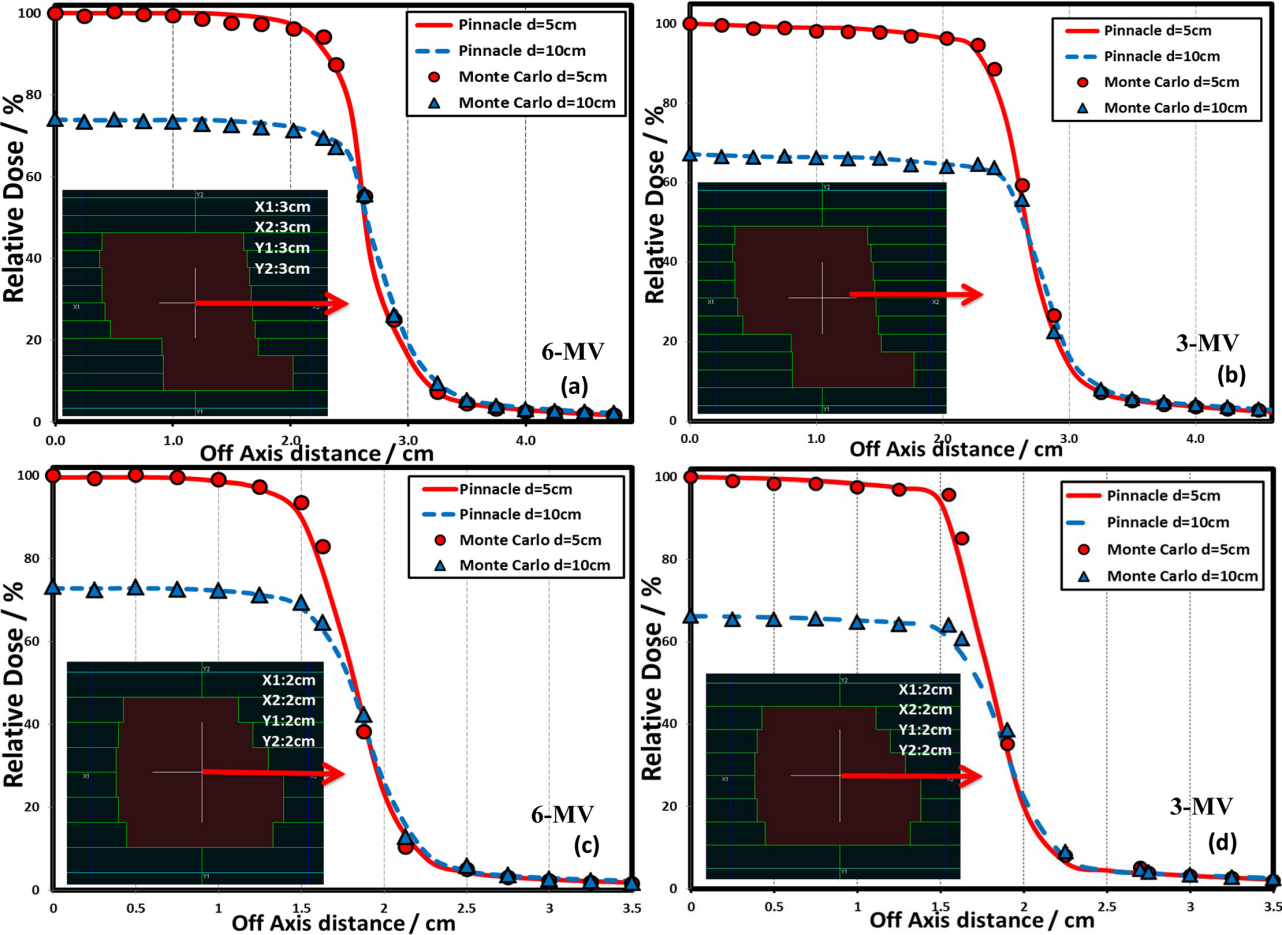
Comparison of Pinnacle^3^ calculated and Monte Carlo simulated dose profiles of 6-MV (a,c) and 3-MV (b, d) beam along the line for two MLC shaped fields (inserts, not to the scale) at 5 and 10 cm depths of a homogeneous water phantom. All doses were normalized to the central axis at 5 cm depth for comparison.

**Fig 3:**
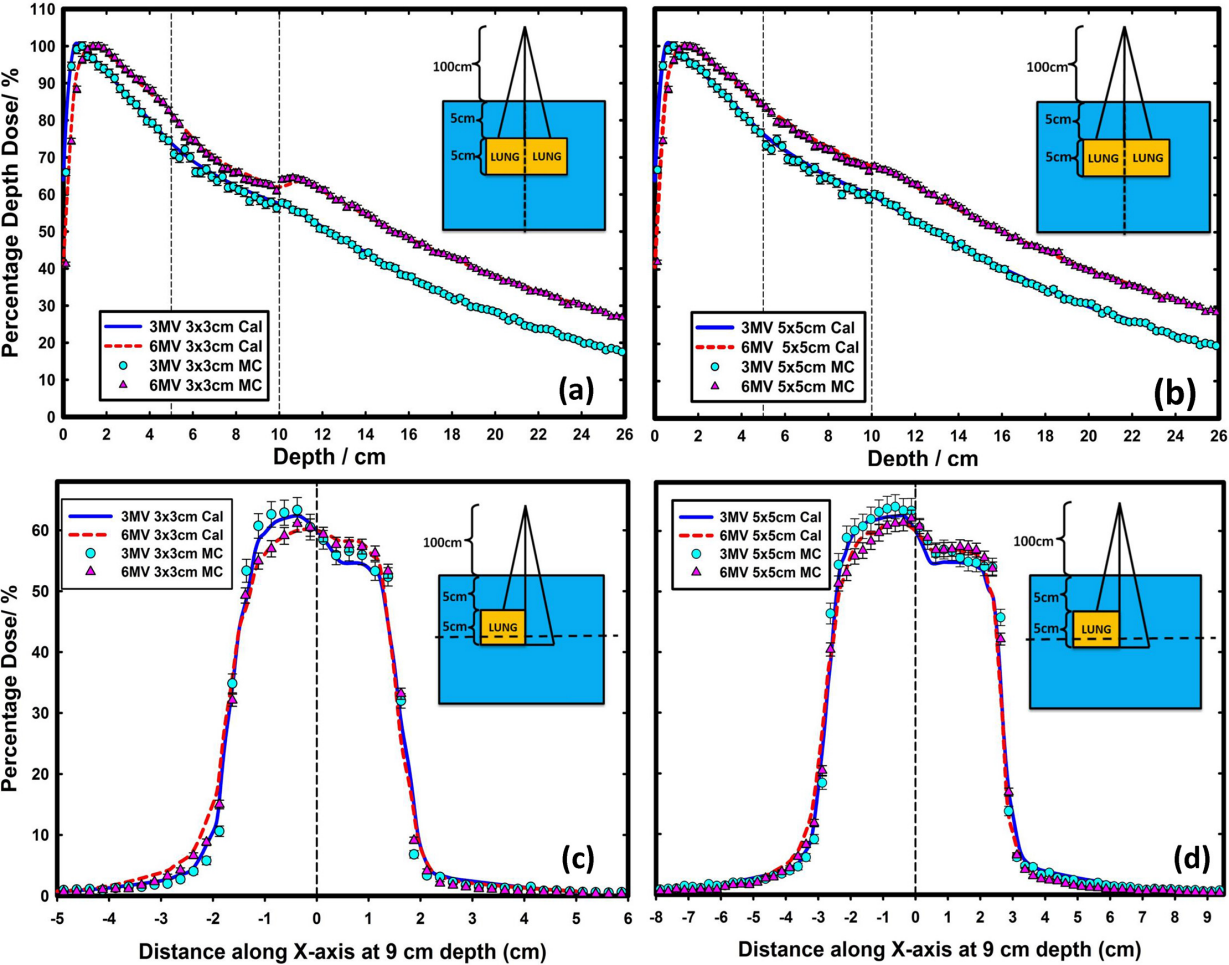
Comparison of Pinnacle^3^ calculated and Monte Carlo simulated percentage depth dose (a, b) and profiles (c, d) of 6-MV and 3-MV beam under inhomogeneous condition. A lung block of 5 cm thick, with a density of 0.3 g/cm^3^ was inserted in a water phantom from 5 cm depth, while the lateral dimensions were either 15×15 cm^2^ or 15×7.5 cm^2^. The field sizes were 3×3 cm^2^ and 5×5 cm^2^.

The Monte Carlo-simulated dose profiles and output factors of the 3-MV photon beams were then commissioned into a Pinnacle^3^ treatment planning system (TPS) version 9.6 (Philips Radiation Oncology Systems, Milpitas, CA). The auto-modeling in Pinnacle^3^ TPS was first used and manual adjustments were then made to ensure that agreement between the Pinnacle^3^ calculations and the Monte Carlo simulations was better than 2%/2mm. The accuracy of the 3-MV beam model commissioned in Pinnacle^3^ TPS has been evaluated by comparing the Pinnacle^3^ dose calculations with Monte Carlo simulations in a variety of beam configurations including both homogeneous water phantom (Figs 1 and 2) and inhomogeneous water phantom with lung block (Fig 3).

### Patient characteristics

31 lung cancer patients were included in this comparative study with institutional review board (IRB) application approved by Yale University Human Investigation Committee (#1404013787). The patients’ characteristics have been summarized in Table 1.

**Table 1:** Summary of patient characteristics.

Characteristic	Median (Range) or No. (%)
**Total number of patients**	31
**PTV Volume (cm** ^**3**^ **)**	38.6 (14.2–188.2)
**Number of Beams**	7–12
**Dose fractionation**	
12.5 Gy × 4	10 (32%)
18 Gy × 3	9 (29%)
10 Gy × 4	4 (13%)
10 Gy × 5	8 (26%)
**Average effective path length (AEP)** ^**a**^ **(cm)**	8.3 (5.6–17.6)
SEP ^b^	7.0 (5.6–9.5)
Patients count	21 (68%)
LEP ^c^	12.1 (10.2–17.6)
Patients count	10 (32%)
**Tumor location**	
Central ^d^	9 (29%)
Peripheral	22(71%)
Upper ^e^	17(55%)
Lower ^f^	14(45%)

^a^ Average effective path length (AEP): a water-equivalent mean path length of all beams from beam entrance to the isocenter.

^b^ SEP: short effective path length, i.e., AEP < 10 cm.

^c^ LEP: long effective path length, i.e., AEP > 10 cm.

^d^Central tumor: tumors within 2 cm in all directions around the proximal bronchial tree per Radiation Therapy Oncology Group (RTOG) 0915.

^e|^ Upper tumor: including right upper lobe (RUL), and left upper lobe (LUL).

^f^ Lower tumor: including right lower lobe (RLL), and left lower lobe (LLL).

### Treatment planning

For all 31 patients, 4DCT scans were performed with Varian real-time position management system (RPM) v1.7.5 and CIVCO Body Pro-Lok immobilization device (CIVCO Medical Solutions, Coralville, Iowa). The 4DCTs were transferred to GE Sim MD workstation to contour the internal target volume (ITV) and 7 mm margin was then added to create the planning target volume (PTV). The average intensity projection (AIP) CT dataset was used for contouring of all relevant OARs including the spinal cord, the trachea, the esophagus and the heart. Three treatment plans were generated for each patient, i.e., 3-MV only, 6-MV only, and dual energy of 3- and 6-MV with the commissioned beam models in Pinnacle^3^. The energies for the dual energy plan were selected based on the effective path length from the beam entrance to the isocenter for each beam. Practically, 3-MV and 6-MV photon beams were mixed almost equally in the dual energy plans.

Identical beam configuration and optimization constraints were used in all three plans for each patient. All plans were optimized such that 100% prescription dose volume covered at least 95% of PTV. The planning optimization constraints for OARs have been used (Table 2). While TG101 and RTOG 0915 guidelines were used as references, optimization constraints were slightly adjusted in the clinic to allow for personalized treatment planning for each individual patient.

**Table 2:** Guidelines of dose volume constraints for the organs-at-risk (OARs)

Organ	Constraints	
Lung (Total lung—ITV)	V_20Gy_	< 7%
	Mean	< 7 Gy
Spinal Cord	18 Gy	< 1 cm^3^
	12 Gy	< 10 cm^3^
Esophagus	35 Gy	< 1 cm^3^
	30 Gy	< 10 cm^3^
Brachial plexus	Point max	< 26 Gy
Heart/Great vessels	40 Gy	< 1 cm^3^
	35 Gy	< 10 cm^3^
Tracheobronchial tree	40 Gy	< 1 cm^3^
	35 Gy	< 10 cm^3^
Skin	Point max	< 30 Gy
Chest wall	30 Gy	< 30cm^3^
	60 Gy	< 1 cm^3^

During the planning, avoidance ring structures were created to facilitate rapid dose fall-off away from the PTV and to restrict the entrance dose of individual beams. IMRT inverse planning was done using direct machine parameter optimization (DMPO) [19]. The final dose distribution was calculated with a collapsed cone convolution (CCC) algorithm on a dose grid of 0.25 cm resolution [20].

### Plan evaluation

Per AAPM TG101 recommendations, CI_100%_, R_50%_ and R_20%_, defined as the ratios of volumes receiving 100%, 50% and 20% of prescribed dose to the PTV volume respectively, were used to quantify the plan quality[21]. Homogeneity index (HI), defined as the ratio of highest dose received by 5% of PTV to lowest dose received by 95% of PTV, was used to evaluate the dose heterogeneity inside the PTV [22,23]

Besides the plan quality indices, D_5%_, D_1%_ and mean dose to the spinal cord, the trachea, the esophagus, the heart and the skin were compared, where D_5%_ and D_1%_ were the doses to at least 5% and 1% of the organ volume, representing the highest doses received by the OARs. For lung tissues, the percent volume receiving 20 Gy (V_20Gy_) and 5 Gy (V_5Gy_), and mean lung dose (MLD) were recorded. The mean doses to each lobe, ipsilateral and contralateral lungs were also compared. A two-tailed t-test was applied in statistical analysis. A significant difference was assumed when p is equal to or less than 0.05.

## Results

### Virtual Linac beam modeling and validation

The 3-MV virtual Linac model was compared with the Monte Carlo simulations in Fig 1. As shown in Fig 1(A), the depth dose curves for the fields of 3 × 3 cm^2^, 10 × 10 cm^2^ and 20 × 20 cm^2^ at 100 cm source-to-surface distance (SSD) were compared between the Pinnacle^3^ predictions and the Monte Carlo simulations. The deviations between the two were shown in the lower part of Fig 1(A), with less than 1% for all the points except in the build-up region where up to 5.5% deviation was observed for the field of 3 × 3 cm^2^. Fig 1(B) showed the lateral dose profile comparisons at 5 cm depth for the same three fields with better than 2%/2 mm agreement. Furthermore, the dose profile comparisons at 5 and 10 cm depths for 100 cm SSD for various irregular fields collimated by the multi-leaf collimator (MLC) were found to be within 2%/2 mm between the Pinnacle^3^ predictions and the Monte Carlo simulations for both the 6-MV and the 3-MV beams (Fig 2). The beam models were also evaluated under inhomogeneous conditions. A lung block of 5 cm thick, with a density of 0.3 g/cm^3^ was inserted in a water phantom from 5 cm depth, while the lateral dimensions were either 15 × 15 cm^2^ or 15 × 7.5 cm^2^. The PDD and profiles for field sizes of 3 × 3 cm^2^ and 5 × 5 cm^2^ at the locations marked with dashed lines in the inserts in Fig 3 were extracted for comparison. The comparison indicated a better than 2%/2 mm agreement between the Pinnacle^3^ predictions and the Monte Carlo simulations at water/lung interface.

### PTV coverage

**Fig 4:**
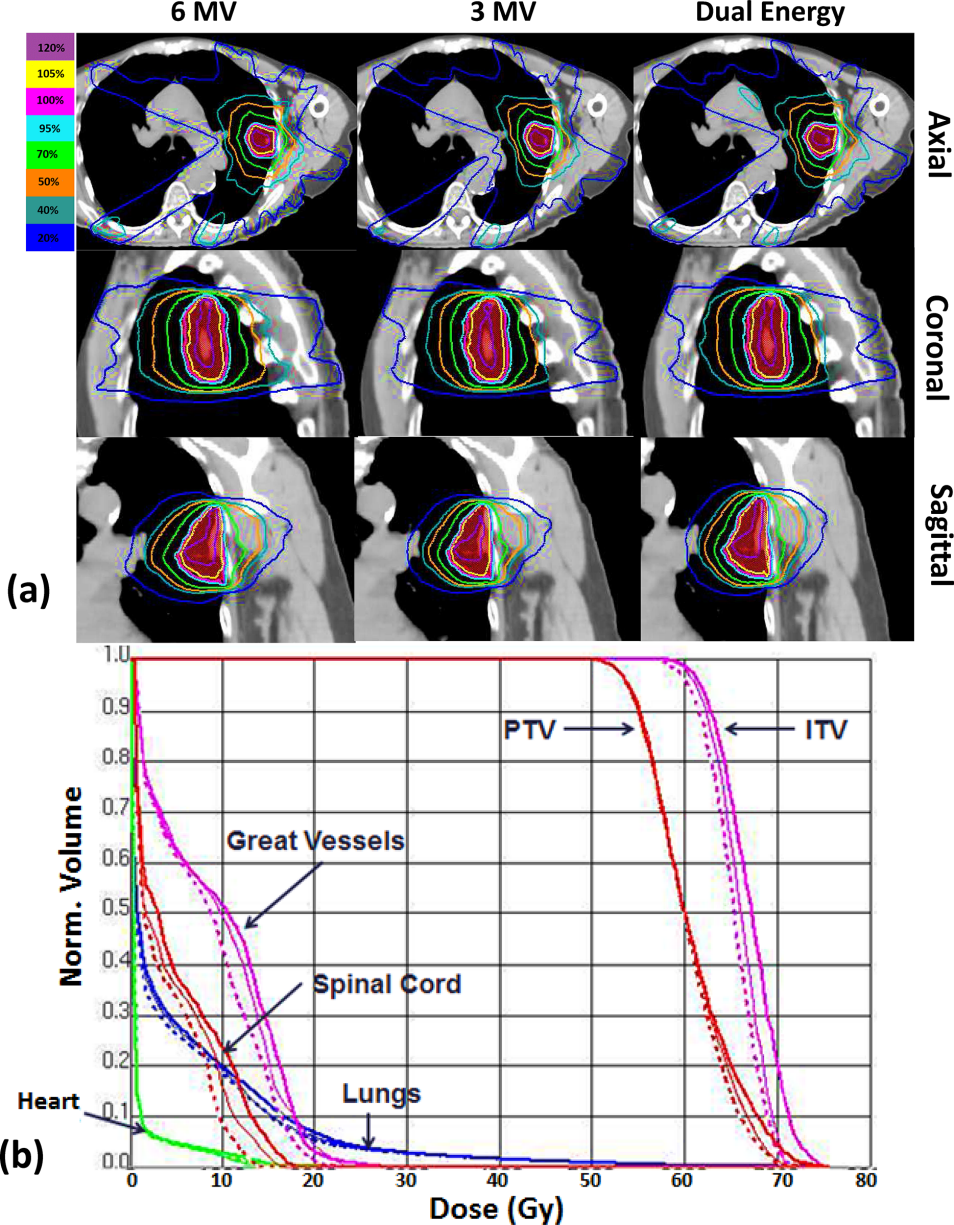
The dose distributions (a) and dose-volume histograms (DVHs) (b) of a representative case. (a) From left to right: 6-MV, 3-MV and the dual energy plans. From top to bottom: axial: sagittal, and coronal views. The isodose lines are 120% (purple), 105% (yellow), 100% (pink), 95% (light blue), 70% (green), 50% (orange), 40% (steel blue) and 20% (dark blue). Notable differences are marked by red arrows. (b) The 6-MV, 3-MV and dual energy plans are shown with thick lines, dashed lines and thin lines, respectively. The DVH curves for trachea, esophagus and skin are not shown due to very low dose level.

The isodose distributions and dose volume histograms (DVHs) of three plans for a peripheral lung tumor were shown in Fig 4 as an example. While all the plans met the conformity requirements, the 3-MV plan (dashed lines) offered the best OAR sparing compared to the 6-MV (thick lines) and the dual energy plans (thin lines) as indicated by the DVHs, and tighter dose envelop and more rapid dose fall-off around PTV as illustrated by the isodose distributions (notable differences are marked with red arrows).

Based on the average effective path length (AEP), we further classified the 31 patients into two groups: short AEP (SEP, < 10 cm) and large AEP (LEP, > 10 cm). As shown in Table 3, the 3-MV plans achieved better dose conformity in SEP group with lower CI_100%_ (1.07±0.14), R_50%_ (4.68±0.83) and R_20%_ (27.3±8.40) compared with those in the 6-MV plans (p < 0.01). Almost no significant difference was observed between 3-MV plans and dual energy plans on these dose conformity indices. For the PTV dose homogeneity, the 3-MV plans produced the most uniform dose distribution (mean HI = 1.26, p < 0.001) at the expense of lowest PTV mean dose (p < 0.001), followed by the dual energy plans (mean HI = 1.28) and the 6-MV plans (mean HI = 1.30).

**Table 3:** Dosimetric comparison of tumor target coverage.

		6 MV		3 MV		Dual Energy		P value		
		Mean±SD	Range	Mean±SD	Range	Mean±SD	Range	6X vs 3X	6X vs Dual	3X vs Dual
CI_100_ ^a^	SEP ^e^	1.09±0.14	(0.94–1.42)	1.07±0.14	(0.91–1.43)	1.07±0.13	(0.92–1.42)	0.01	0.07	0.49
	LEP ^f^	1.09±0.10	(0.99–1.28)	1.11±0.15	(0.97–1.40)	1.09±0.13	(0.96–1.30)	0.10	0.41	0.09
R_50%_ ^b^	SEP	4.90±0.93	(3.17–6.17)	4.68±0.83	(3.15–6.27)	4.81±0.92	(3.09–6.34)	< 0.001	0.04	0.02
	LEP	4.66±0.71	(3.75–6.14)	4.98±0.95	(3.71–6.10)	4.56±0.61	(3.76–5.35)	0.05	0.26	< 0.05
R_20%_ ^c^	SEP	28.1±9.30	(9.2–47.1)	27.3±8.40	(9.0–42.6)	27.2±8.80	(8.7–44.1)	0.01	0.01	0.38
	LEP	34.3±11.3	(16.5–50.1)	36.2±12.0	(18.1–54.2)	34.5±11.3	(16.8–50.8)	0.02	0.44	< 0.001
HI^d^	SEP	1.30±0.08	(1.12–1.46)	1.26±0.06	(1.14–1.36)	1.28±0.07	(1.11–1.42)	< 0.001	< 0.001	0.06
	LEP	1.27±0.09	(1.10–1.37)	1.23±0.09	(1.10–1.41)	1.25±0.09	(1.10–1.41)	0.01	0.07	0.02
PTV D_mean_(Gy)	SEP	59.1±3.50	(50.6–63.7)	58.4±3.60	(48.5–66.1)	58.6±3.08	(49.5–65.5)	< 0.001	0.04	0.14
	LEP	53.4±6.10	(44.7–62.5)	52.9±6.50	(44.1–63.5)	53.2±6.01	(44.4–63.2)	0.06	0.19	0.07
ITV D_mean_(Gy)	SEP	64.4±5.10	(54.3–65.7)	62.8±4.50	(52.7–71.3)	63.5±4.08	(54.2–70.7)	< 0.001	< 0.001	0.03
	LEP	57.0±6.50	(47.1–65.6)	55.9±6.90	(45.0–66.8)	55.6±6.05	(46.5–66.7)	0.01	0.08	0.02

^a^CI_100%_: ratio of prescription isodose volume to PTV volume.

^b^ R_50%_: ratio of the volume of the 50% prescription isodose curve to PTV volume.

^c^R_20%_: ratio of the volume of the 20% prescription isodose curve to PTV volume.

^d^ HI, Heterogeneity Index: ratio of highest dose received by 5% of PTV to lowest dose received by 95% of PTV.

^e^ SEP: short effective path length, i.e., average effective path length (AEP) is less than 10 cm.

^f^ LEP: long effective path length, i.e., average effective path length (AEP) is larger than 10 cm.

Within LEP group of 10 thick patients, the dual energy plans showed slightly better dose conformity with lowest CI_100%_ (1.09±0.13), R_50%_ (4.56±0.61) and R_20%_ (34.5±11.3) compared with those in the 6-MV plans. The 3-MV plans still provided the most homogeneous doses in the PTV (HI = 1.23) with lower ITV and PTV mean dose, due to weaker penetration power of the 3-MV photons.

### OAR doses

**Fig 5:**
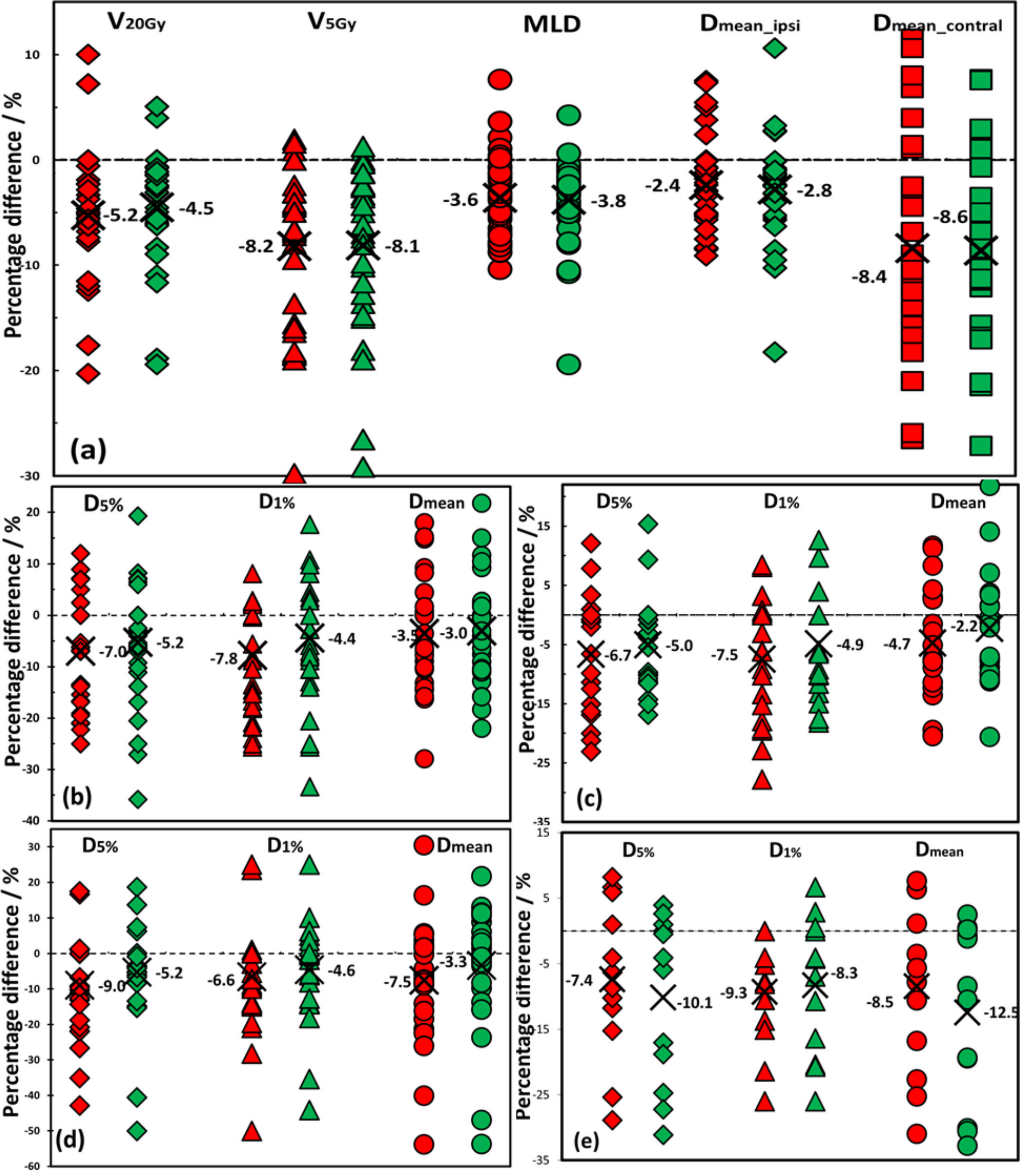
The percentage dosimetric differences for the lung (a), cord (b), esophagus (c), trachea (d), and the heart (e). The percentage difference was calculated as $\frac{D_{3MV/dual}-D_{6MV}}{D_{6MV}}$. Each patient is illustrated by a red symbol (left) for 3-MV and green one (right) for dual energy plan. The mean difference of each index for the whole group is marked with a cross x.

The dosimetric indices of OARs for all 31 patients are shown in Fig 5. In general, the 3-MV plans offered significantly better sparing of the normal tissues compared to the 6-MV plans as indicated by the reduction of the dose indices for various OARs.

**Fig 6:**
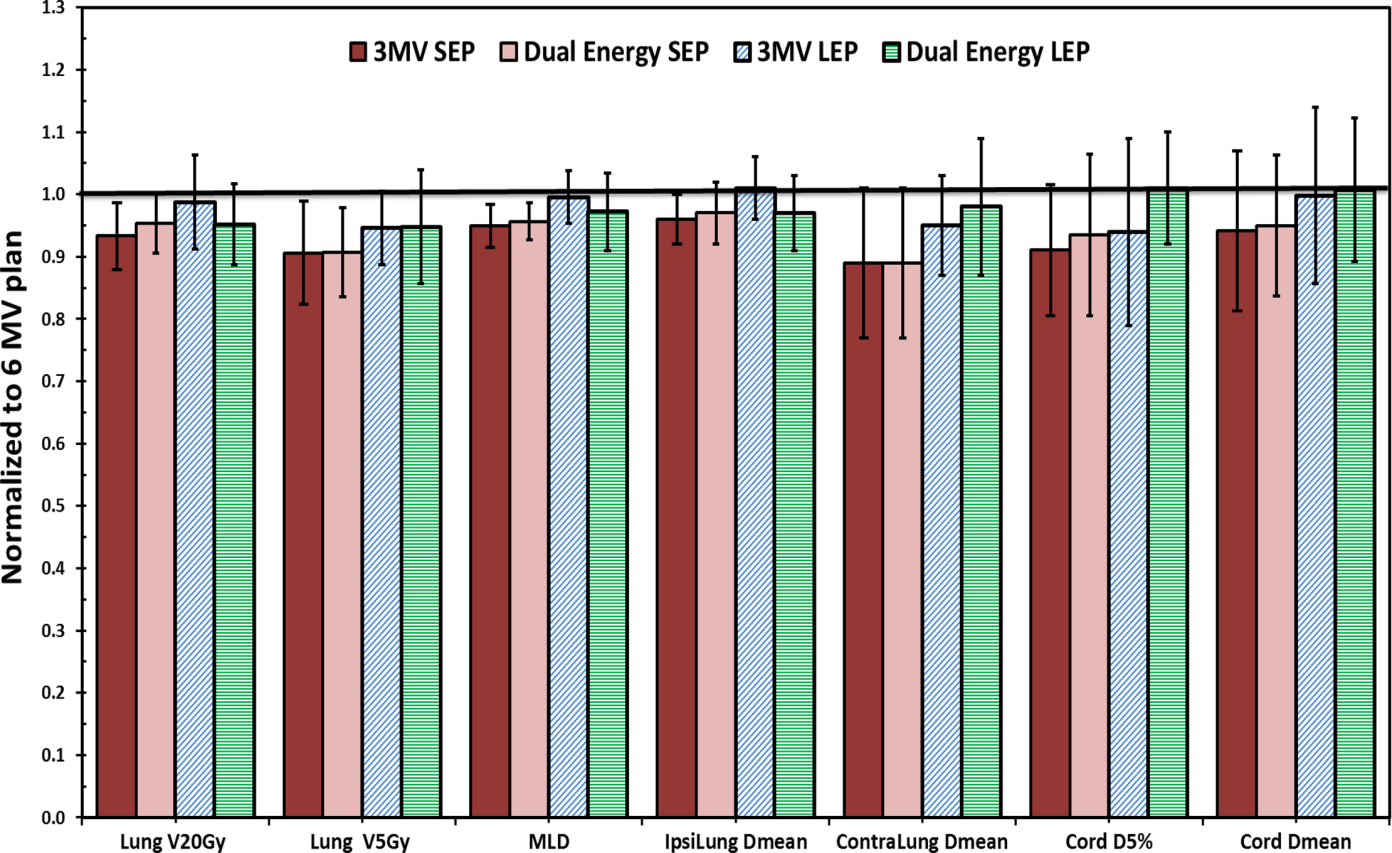
Dosimetric comparisons of lung and spinal cord. All parameters were normalized to the counterparts of 6-MV plan shown as the solid black line. Patients were categorized into short effective path length (SEP, < 10 cm) and Long effective path length (LEP, > 10 cm) groups.

Fig 5(A) showed the percentage differences of the lung indices for the 3-MV and dual energy plans compared to the 6-MV plans. Large variations were observed among 31 patients. The mean reductions for V_20Gy_ and V_5Gy_ were 5.2% and 8.2%, and 4.5% and 8.1% for the 3-MV and the dual energy plans, respectively (p < 0.05), as marked with a symbol of X in Fig 5(A). Comparable MLDs were observed for all three types of plans. A majority of the patients benefited from the contralateral lung sparing and the mean dose reduction of contralateral lung were 8.4% and 8.6% for the 3-MV and dual energy plans, respectively (p < 0.001). More detailed dosimetric comparisons are shown in Fig 6 for the lung and the spinal cord. For SEP group, the 3-MV and dual energy plans were almost identical in terms of lung dosimetric indices. The average/maximum reductions of V_20Gy_, V_5Gy_ and MLD of the lung were 7%/20%, 9%/30% and 5%/10%, respectively in the 3-MV plans (all p < 0.05). On average, the contralateral lung received 11% less dose in SEP group. For LEP group, the dual energy plans showed slightly superior results in lung dose reduction.

In terms of cord dose, compared to the 6-MV plans, the 3-MV and the dual energy plans delivered 7.0% and 5.2% less doses to D_5%_, and 7.8% and 4.4% less doses to D_1%_ of the spinal cord, respectively, as shown in Fig 5(B). Meanwhile, the mean cord dose was 3.5% and 3% lower in the 3-MV and dual energy plans, respectively (all p < 0.05). As shown in Fig 6, in SEP group, the 3-MV and dual energy plans performed equally well with 9% reduction of D_5%_ and more than 5% reduction in mean dose of cord (p < 0.05). In LEP group, no significant difference was observed among three types of plans in terms of cord dose distributions (p > 0.05).

For the esophagus and trachea, both the 3-MV and the dual energy plans yielded reduced mean dose and decreased D_5%_ and D_1%_ as shown in Fig 5(C) and 5(D). For all patients, the D_5%_ and D_1%_ of the esophagus were reduced by 6.7% and 7.5% for the 3-MV and 5.0% and 4.9% for the dual energy plans respectively, as compared to the 6-MV plans. For the trachea, the D_5%_ and D_1%_ reductions were 9.0% and 6.6% for the 3-MV and 5.2% and 4.6% for the dual energy plans, respectively. In SEP group, significant sparing was observed in the 3-MV plans as 10.6% and 11.4% D_5%_ reductions (p<0.05) for the esophagus and trachea, respectively, while almost identical mean doses were observed in LEP group (p > 0.05).

For the 13 patients with non-negligible heart dose (mean heart dose > 0.7 Gy), both 3-MV and dual energy plans showed better heart sparing. Compared to the 6-MV plans, the D_5%_ and D_1%_ reductions were 7.4% and 9.3% for the 3-MV and 10.1% and 8.3% for the dual energy plans, respectively (p < 0.05). The mean heart dose varied from 1.1 to 12.2 Gy in the 6-MV plans depending on tumor locations, and they were 0.78 to 12.1 Gy in the 3-MV plans and 0.76 to 12.2 Gy in the dual energy plans. On average, the 3-MV and dual energy plans spared the mean heart doses by 8.5% and 12.5%, respectively (p < 0.05).

### Integral dose and delivery efficiency

**Fig 7:**
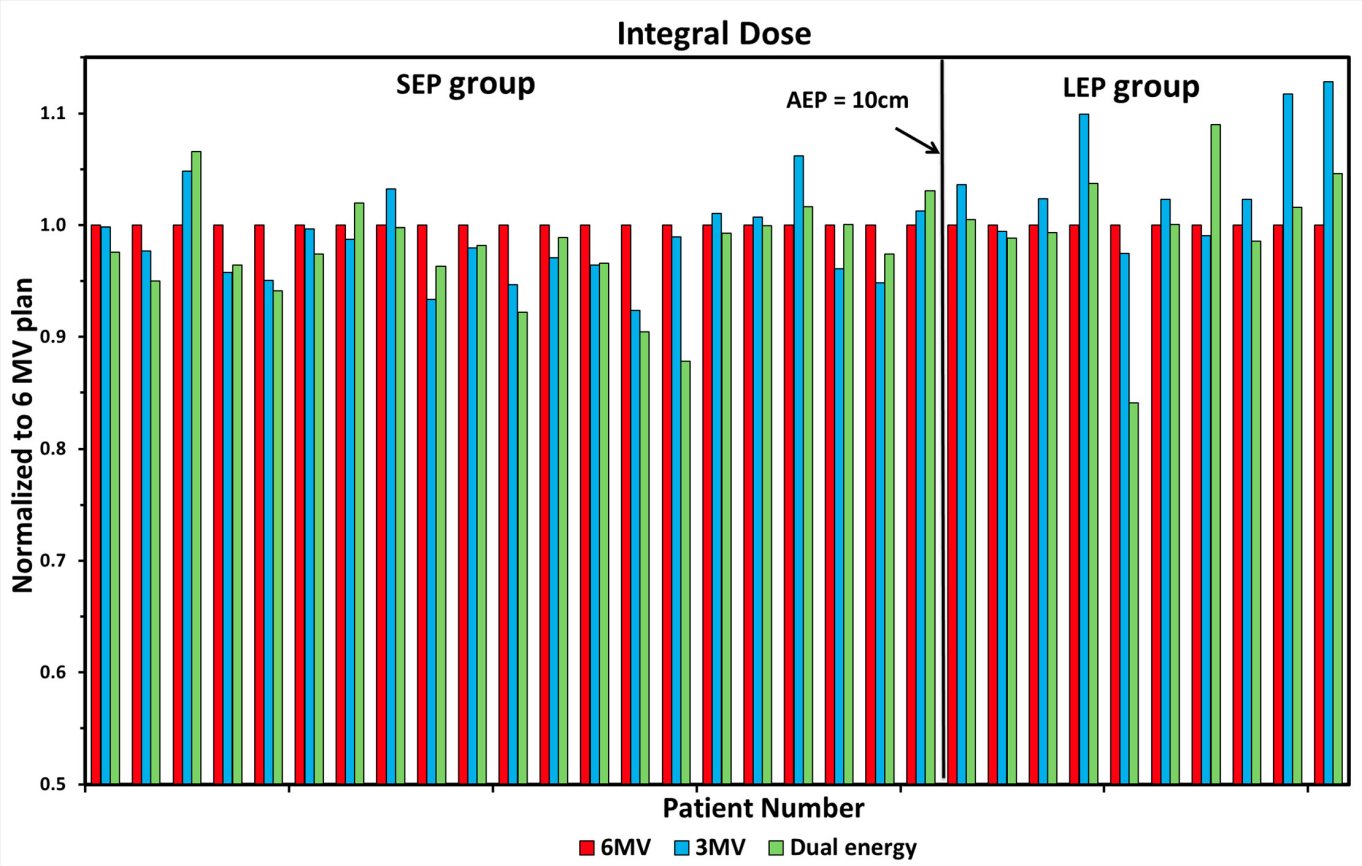
Comparison of integral dose for three plans. Individual patients were represented with the average effective path length (AEP) and categorzed into two groups (short effective path legnth (SEP) and long effective path legnth (LEP)). Integral doses were calcuated as *D*_*meanBody*_ × *V*_*Body*_ − *D*_*meanPTV*_ × *V*_*PTV*_ and normalized to 6-MV plans.

Integral dose, defined as the volume integral of the dose deposited in the patient anatomy with PTV excluded, was compared in Fig 7 for all the 31 patients. Only 3 out of 31 patients received more than 5% higher integral dose in the 3-MV plans as compared to the 6-MV plans, all of whom were thick patients with long average effective path length (> 10 cm). For other patients, the integral dose was comparable to or even lower than the 6-MV plans (p < 0.05). The results also showed that for thick patients in LEP group, the 6-MV plans were more preferable with less integral dose, followed by the dual energy plans.

**Fig 8:**
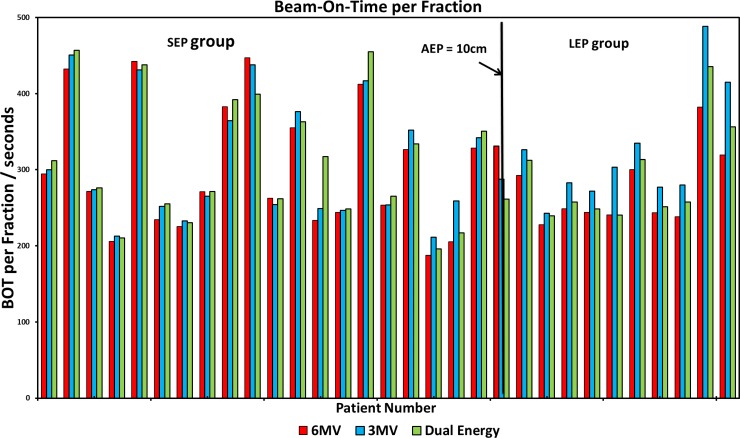
Comparison of Beam-On-Time for three plans. Individual patients were represented with the average effective path length (AEP) and categorized into two groups (short effective path legnth (SEP) and long effective path legnth (LEP)).

The beam-on time (BOT), calculated as the total monitor units (MU) per fraction divided by the dose rate of 600 MU/minute commonly used in SBRT, were used to evaluate the delivery efficiency. As shown in Fig 8, the BOTs were similar among three plans for thin patients (SEP). For thick patients (LEP), the 3-MV plans required the longest beam-on-time to deliver the prescription doses.

## Discussion

In this work, we studied the dosimetric effects of 3-MV photon beams for lung SBRT treatments. Compared to the 6-MV plans, the 3-MV plans showed better target dose conformity and homogeneity (Fig 4 and Table 3) as well as better sparing of the OARs (Figs 5 and 6) for the thin lung cancer patients. For thick patients, 3-MV photon beams were shown less beneficial due to their weaker penetration power, while those thick patients could benefit from the dual energy plans with comparable PTV coverage, integral dose and reduced dose to the critical structures.

Previous studies have demonstrated that intermediate energy photons have potential dosimetric benefits for intracranial stereotactic radiosurgery (1.2-MV) [5] and extracranial robotic IMRT (2-MV/6-MV) [4]. Specifically, Dong *et al*. investigated the feasibility of using 2-/6-MV photons for extracranial robotic IMRT treatments of a variety of lesions with one patient per lesion site [4]. In their study on lung IMRT treatment, Dong *et al*. showed that 2-MV photons can reduce the V_20Gy_, V_5Gy_ and mean dose of lung tissues by 13%, 30% and 24%, respectively, compared to 6-MV photons. In this work, we investigated the effects of 3-MV photons on Linac-based SBRT treatments of 31 lung cancer patients. With 31 lung cancer patients covering a full range of clinical conditions in terms of patient size, age, gender, tumor volume, tumor location and tumor laterality, we were able to summarize the dosimetric variations with statistical significance for plan comparison. Overall, our results indicate that on average, 3-MV photons can reduce the V_20Gy_, V_5Gy_ and MLD by 5.2%, 8.2% and 3.6%, respectively, in comparison to 6-MV photons. In addition, for thin patients with short effective path length (< 10 cm), the average/maximum reductions of the V_20Gy_, V_5Gy_ and MLD were 7%/20%, 9%/30% and 5%/10%, respectively in the 3-MV plans. The large disparity between Dong *et al*.’s results and our results may largely be due to the sample size and different energy of photons being used. However, both studies have confirmed more conformal target coverage, better homogeneity and better OAR sparing in lung IMRT treatments with intermediate energy photons.

The lower penetration power and higher skin dose are thought to be the limitations of intermediate energy photon beams. However, with modulated dose delivery, it has been shown that radiotherapy has become less restricted by these limitations in low energy X-rays [24,25]. This is because as photon beams go through patient anatomy from multiple angles, the dose delivery burden will be largely diluted [26]. In this study, compared to the 6-MV plans, the absolute mean skin dose increased in the range of 46 to 330 mGy and 2 to 152 mGy for the 3-MV and dual energy plans, respectively. The median/maximum values of D1% for the skin were found to be 10.2/21.3 Gy, 12.9/23.0 Gy and 12.4/23.0 Gy for the 6-MV, 3-MV and dual energy plans, respectively, which were still much lower than the tolerance (maximum dose < 30 Gy). Thus, skin dose will not be a serious concern in the adoption of intermediate energy photon beams for clinical applications.

Due to rapid dose fall-off with small penumbra at the field edge, 3-MV photon beams can help reduce the doses deposited to the adjacent critical structures in lung cancer treatments as shown in this study. The clinical benefits can be multi-folds. First, our study indicated that 3-MV photons can on average reduce V_20Gy_ and V_5Gy_ by 7% and 9%, respectively compared to 6-MV photons. Reduced V_20Gy_ and V_5Gy_ can potentially reduce the risk of radiation pneumonitis and pulmonary fibrosis, which could compromise the patient’s quality of life [27]. Second, in this study, 3-MV photons were shown to reduce D_5%_ of the cord, trachea and esophagus by 6.7 to 9% and reduce D_1%_ by 6.6 to 7.8% as compared to 6-MV photons. As the spinal cord, trachea and esophagus are serial organs, even a small volume irradiated beyond its threshold can potentially lead to whole organ failure [28]. Hence, it is very important to reduce the high doses to these critical organs. Third, when the lung tumor is proximal to the heart, a fraction of heart volume could receive a relatively high dose which raises potential risk of radiation-related heart diseases [29,30]. Our study indicated that the 3-MV plans outperformed the 6-MV plans with an average of 7.4% D_5%_ reduction (p < 0.05) and 8.5% mean dose reduction to the heart (p < 0.05), which translated into 0.72 Gy and 0.35 Gy in absolute dose reduction in D_5%_ and mean heart dose, respectively.

Another potential benefit of using 3-MV photon beams could be in the treatment of pediatric cancer patients who have relatively small dimensions with close proximity between the tumor and the critical structures. As children are far more susceptible to radiation induced secondary malignancies than adults, the radiation induced toxicities to pediatric cancer patients have been actively investigated in the past 50 years [31]. In this study, the considerable improvement in target coverage, target homogeneity and OAR sparing related to 3-MV photon beams in thin lung cancer patients implies that intermediate energy photon beams such as 3-MV photons could be a better choice for radiation treatments of pediatric cancer patients. Further study will be needed to explore the role of intermediate energy photons in the radiotherapeutic management of pediatric cancers.

It has been shown that MV fan beam CT (MVCT) with effective energy of 3.5-MV from a Helical Tomotherapy unit can provide sufficient contrast for soft-tissue delineation [32,33]. Several studies have further shown that with low-Z targets in linear accelerators producing photon beams at 2.35-MV and 1.9-MV, the image quality could be greatly enhanced as compared to the 6-MV photons [34,35]. In fact, 2.5-MV photon beams from a Varian TrueBeam linac have been available in the clinic for routine portal imaging with better image quality than conventional 6-MV photons. Hence, it is likely that a single intermediate energy photon beam can be used for both radiation treatments and image guidance concurrently for certain applicable situations such as lung cancer treatments and pediatric patients. We will report our investigation results on this topic in our future communications.

## Conclusion

Compared to 6-MV photon beams, 3-MV photon beams have statistically significant dosimetric benefits in treating lung tumors in terms of improved tumor coverage and reduced doses to the adjacent critical structures. Intermediate energy photons (<6-MV) could be considered and added into current radiotherapy arsenal to reduce the radiation-related toxicities while maintaining sufficient tumor control in lung cancer radiotherapy.

## Supporting Information

S1 Table The percentage depth dose (PDD) data for the 3-MV photon beam. The Monte Carlo simulated results, Pinnacle^3^ calculation results and the deviations for three different field sizes (3×3 cm^2^, 10×10 cm^2^ and 20×20 cm^2^) were listed. These were the original data for Fig 1(A).
(XLSX)

S2 Table The lateral dose profiles data for the 3-MV photon beam. The Monte Carlo simulated results and Pinnacle^3^ calculation results for three different field sizes (3×3 cm^2^, 10×10 cm^2^ and 20×20 cm^2^) were listed. These were the original data for Fig 1(B).
(XLSX)
